# Supplementary material for: Smooth Interpolating Curves with Local Control and Monotone Alternating Curvature
Source: Comput Graph Forum. 2022 Oct 6;41(5):25–38. doi: 10.1111/cgf.14600 (PMC9827861; doi:10.1111/cgf.14600)
Supplement: Supplementary file 1 — Supplement Material [file CGF-41-25-s001.zip › Local-Smooth-Interpolating-MonoCurvature/extern/clothoids/submodules/Utils/docs/api-cpp/program_listing_file_Utils_Console.hxx.html]

Program Listing for File Console.hxx — Utils v1.2

### Navigation

- index
- toc
- Utils »
- Program Listing for File Console.hxx

# Program Listing for File Console.hxx¶

↰ Return to documentation for file (`Utils/Console.hxx`)

```
/*--------------------------------------------------------------------------*\
 |                                                                          |
 |  Copyright (C) 2020                                                      |
 |                                                                          |
 |         , __                 , __                                        |
 |        /|/  \               /|/  \                                       |
 |         | __/ _   ,_         | __/ _   ,_                                |
 |         |   \|/  /  |  |   | |   \|/  /  |  |   |                        |
 |         |(__/|__/   |_/ \_/|/|(__/|__/   |_/ \_/|/                       |
 |                           /|                   /|                        |
 |                           \|                   \|                        |
 |                                                                          |
 |      Enrico Bertolazzi                                                   |
 |      Dipartimento di Ingegneria Industriale                              |
 |      Universita` degli Studi di Trento                                   |
 |      email: enrico.bertolazzi@unitn.it                                   |
 |                                                                          |
\*--------------------------------------------------------------------------*/


#pragma once

#ifndef DOXYGEN_SHOULD_SKIP_THIS
#ifndef UTILS_CONSOLE_dot_HXX
#define UTILS_CONSOLE_dot_HXX
#endif

namespace Utils {

  #ifndef DOXYGEN_SHOULD_SKIP_THIS
  using std::string;
  using std::mutex;
  using std::basic_istream;
  using std::basic_ostream;
  using std::cout;
  #endif

  typedef basic_istream<char> istream_type;
  typedef basic_ostream<char> ostream_type;

  string basename( char const * const filename );

  class Console {

    mutable mutex m_message_mutex; // mutex for critical section

  #ifndef DOXYGEN_SHOULD_SKIP_THIS
  public:
    class Console_style {
    public:
      rang::style s;
      rang::fg    f;
      rang::bg    b;
    };
  #endif

  private:

    ostream_type * m_stream;

    // 0 only fatal, error
    // 1 + warning
    // 2
    int m_level;

    Console_style m_message_style;
    Console_style m_warning_style;
    Console_style m_error_style;
    Console_style m_fatal_style;

    Console() = delete;
    Console( Console const & ) = delete;

  public:

    Console( ostream_type * stream = &cout, int level = 4 );

    void change_level( int new_level );
    void change_stream( ostream_type * new_stream );
    int  get_level() const { return m_level; }

    ostream_type * get_stream() const { return m_stream; }

    void changeLevel( int new_level ) { this->change_level(new_level); }
    void changeStream( ostream_type * new_stream ) { this->change_stream( new_stream ); }
    int  getLevel() const { return m_level; }

    ostream_type * getStream() const { return m_stream; }

    Console const & flush() const { m_stream->flush(); return *this; }

    Console const &
    message( string const & msg, int msg_level = 4 ) const;

    Console const &
    semaphore( unsigned ryg, string const & msg, int msg_level = 0 ) const;

    Console const &
    warning( string const & msg ) const; // level >= 2

    Console const &
    error( string const & msg ) const; // level >= 1

    Console const &
    fatal( string const & msg ) const; // level >= 0

    Console const & black   ( string const & msg, int msg_level = 0 ) const;
    Console const & red     ( string const & msg, int msg_level = 0 ) const;
    Console const & green   ( string const & msg, int msg_level = 0 ) const;
    Console const & yellow  ( string const & msg, int msg_level = 0 ) const;
    Console const & blue    ( string const & msg, int msg_level = 0 ) const;
    Console const & magenta ( string const & msg, int msg_level = 0 ) const;
    Console const & cyan    ( string const & msg, int msg_level = 0 ) const;
    Console const & gray    ( string const & msg, int msg_level = 0 ) const;

    Console const & black_reversed   ( string const & msg, int msg_level = 0 ) const;
    Console const & red_reversed     ( string const & msg, int msg_level = 0 ) const;
    Console const & green_reversed   ( string const & msg, int msg_level = 0 ) const;
    Console const & yellow_reversed  ( string const & msg, int msg_level = 0 ) const;
    Console const & blue_reversed    ( string const & msg, int msg_level = 0 ) const;
    Console const & magenta_reversed ( string const & msg, int msg_level = 0 ) const;
    Console const & cyan_reversed    ( string const & msg, int msg_level = 0 ) const;
    Console const & gray_reversed    ( string const & msg, int msg_level = 0 ) const;

    void
    set_message_style(
      rang::style const & s, rang::fg const & f, rang::bg const & b
    ) {
      m_message_style.s = s; m_message_style.f = f; m_message_style.b = b;
    }

    void
    setMessageStyle(
      rang::style const & s, rang::fg const & f, rang::bg const & b
    ) {
      this->set_message_style( s, f, b );
    }

    void
    set_warning_style(
      rang::style const & s, rang::fg const & f, rang::bg const & b
    ) {
      m_warning_style.s = s; m_warning_style.f = f; m_warning_style.b = b;
    }

    void
    setWarningStyle(
      rang::style const & s, rang::fg const & f, rang::bg const & b
    ) {
      this->set_warning_style( s, f, b );
    }

    void
    set_error_style(
      rang::style const & s, rang::fg const & f, rang::bg const & b
    ) {
      m_error_style.s = s; m_error_style.f = f; m_error_style.b = b;
    }

    void
    setErrorStyle(
      rang::style const & s, rang::fg const & f, rang::bg const & b
    ) {
      this->set_error_style( s, f, b );
    }

    void
    set_fatal_style(
      rang::style const & s, rang::fg const & f, rang::bg const & b
    ) {
      m_fatal_style.s = s; m_fatal_style.f = f; m_fatal_style.b = b;
    }

    void
    setFatalStyle(
      rang::style const & s, rang::fg const & f, rang::bg const & b
    ) {
      this->set_fatal_style( s, f, b );
    }

    void
    set_off() const {
      #ifndef UTILS_OS_WINDOWS
      rang::setControlMode( rang::control::Off );
      #endif
    }

    void setOff() const { this->set_off(); }

    void
    set_auto() const {
      #ifdef UTILS_OS_WINDOWS
      rang::setWinTermMode( rang::winTerm::Auto );
      #else
      rang::setControlMode( rang::control::Auto );
      #endif
    }

    void setAuto() const { this->set_auto(); }
  };

}

#endif
```

### Quick search

### Table of Contents

- rang - Colors for your Terminal
- Zstr
- C++ API

«
hide menu

menu
sidebar
»

### Navigation

- index
- toc
- Utils »
- Program Listing for File Console.hxx

© Copyright 2021, Enrico Bertolazzi.
Created using Sphinx 4.2.0.
